# Assessment of gestational age at antenatal care visits among Kenyan women to inform delivery of a maternal respiratory syncytial virus (RSV) vaccine in low- and middle-income countries

**DOI:** 10.12688/wellcomeopenres.19161.1

**Published:** 2023-04-03

**Authors:** Joyce U. Nyiro, Bryan O. Nyawanda, Elizabeth Bukusi, Marianne W. Mureithi, Nickson Murunga, D. James Nokes, Godfrey Bigogo, Nancy A. Otieno, Victor A. Opere, Alice Ouma, Clint Pecenka, Patrick K. Munywoki

**Affiliations:** 1 Kenya Medical Research Institute-Wellcome Trust Research Programme, Centre for Geographic Medicine Research-Coast, Kilifi, Kenya; 2 Kenya Medical Research Institute, Center for Global Health Research, Kisumu, Kenya; 3 Kenya Medical Research Institute, Centre for Microbiology Research, Nairobi, Kenya; 4 Department of Microbiology, University of Nairobi, Nairobi, Nairobi County, Kenya; 5 School of Life Sciences, University of Warwick, Coventry, England, UK; 6 Center for Vaccine Innovation and Access, PATH, Seattle, Washington, USA

**Keywords:** Pregnant women, Antenatal care, Gestational age, Maternal vaccine, vial size, Prevention, Respiratory Syncytial Virus

## Abstract

**Background: **Maternal respiratory syncytial virus (RSV) vaccines that are likely to be implementable in low- and middle-income countries (LMICs) are in final stages of clinical trials. Data on the number of women presenting for antenatal care (ANC) per day and proportion attending within the proposed gestational window for vaccine delivery, is a prerequisite to guide development of vaccine vial size and inform vaccine uptake in this setting.

**Methods:** We undertook administrative review and abstraction of ANC attendance records from 2019 registers of 24 selected health facilities, stratified by the level of care, from Kilifi, Siaya and Nairobi counties in Kenya. Additional data were obtained from Mother and Child Health (MCH) booklets of women in each of the Health and Demographic Surveillance System (HDSS) areas of Kilifi, Nairobi and Siaya. Data analysis involved descriptive summaries of the number (mean, median) and proportion of women attending ANC within the gestational window period of 28-32 weeks and 24-36 weeks.

**Results:** A total of 62,153 ANC records were abstracted, 33,872 from Kilifi, 19,438 from Siaya and 8,943 from Nairobi Counties. The median (Interquartile range, IQR) number of women attending ANC per day at a gestational age window of 28-32 and 24-36 weeks, respectively, were: 4 (2-6) and 7 (4-12) in dispensaries, 5 (2-9) and 10 (4-19) in health centres and 6 (4-11) and 16 (10-26) in county referral hospitals. In the HDSS areas of Kilifi, Siaya and Nairobi, pregnant women attending at least one ANC visit, within a window of 28-32 weeks, were: 77% (360/470), 75% (590/791) and 67% (547/821), respectively.

**Conclusions:** About 70% of pregnant women across three distinct geographical regions in Kenya, attend ANC within 28-32 weeks of gestation. A multidose vial size with about five doses per vial, approximates daily ANC attendance and would not incur possible wastage in similar settings.

## Introduction

Maternal immunisation is considered as one of the most plausible strategies, to prevent hospitalization with respiratory syncytial virus (RSV) associated lower respiratory tract infection (LRTI) among infants ^1– 3^. Global estimates have shown RSV to be the cause of about 2.9 to 4.6 million hospitalizations among children under 5 years of age ^4^, with severe RSV-associated disease experienced among infants under 6 months of age ^4– 6^. As a result, most preventive interventions for RSV target this young age group.

Developing an infant vaccine that is highly immunogenic has been a challenge due to interference with maternal antibodies ^6, 7^ and reactogenicity experienced during clinical trials with the first formalin inactivated RSV vaccine targeting infants ^8^. In the absence of licensed childhood RSV vaccines, there are other products in advanced stages of development for prevention and these include long-acting RSV-specific monoclonal antibody for paediatric prophylaxis ^9, 10^ and maternal vaccines ^1, 9^.

Several maternal vaccines are in advanced stages of clinical trials (NCT04032093, NCT04424316 and NCT03049488) and have shown promising results ^9^. A subunit RSV F maternal vaccine (NCT04032093) has shown efficacy of 84.7% (95% CI; 21.6-97.6%) in preventing medically attended infant RSV-LRTI in phase IIb clinical trials. The existence of antenatal care (ANC) clinics ^11^; which is a platform widely used for delivery of the maternal tetanus vaccine offer an opportunity for implementation of the new maternal RSV vaccine. To ensure optimal passive transfer of antibodies and maximum benefit to the infant, the maternal RSV vaccines are expected to include administration within a restricted window of gestational age. Consequently, knowledge on gestational age at presentation for ANC services is required to inform the design of the implementation strategy.

Data on ANC coverage and timing has been scarce in low- and middle-income countries (LMICs). Previously, we presented results for gestational age at ANC visit and the potential coverage for maternal RSV vaccination within three proposed gestational age windows for vaccine delivery of 28–32 weeks, 26–33 weeks and 24–36 weeks ^12^. This study was from a single site in Kilifi, Kenya, which showed about 77% of pregnant women attended at least one ANC visit within the proposed vaccination window period of 28–32 weeks of gestation ^12^. However, these results did not provide comprehensive information on how a maternal RSV vaccine will be delivered within a health facility setting *i.e.*, the number of women attending ANC per day and how these will vary within the different levels of health care. The additional information is vital in determining the size of vaccine vials for optimal use in these settings.

To inform vaccine uptake and guide development of vaccine vial size for LMICs, we provide comprehensive data on the number of pregnant women per day and proportion accessing ANC services within two proposed windows of maternal RSV vaccine delivery, 28–32 weeks, and 24–36 weeks of gestation, at various levels of health care in three counties, with well characterised populations in Kenya.

## Methods

### Study sites

The study was conducted in Kilifi, Siaya and Nairobi Counties in Kenya, within the health and demographic surveillance systems (HDSS) areas and within selected health facilities. The Kilifi, Siaya and Nairobi populations have been previously described ^13– 15^. The Kenya Medical Research Institute-Wellcome Trust Research Programme (KEMRI-WTRP) supports the Kilifi HDSS, which was mapped for clinical and epidemiological surveillance since the year 2000 ^13^. In Nairobi and Siaya HDSS, the study was conducted within sites, where there is active longitudinal population based infectious disease surveillance (PBIDS). The Nairobi and Siaya PBIDS cover two villages in Kibera informal settlement in Nairobi County and 33 villages in Asembo, Siaya County since 2006 ^15^. The PBIDS platform is managed by KEMRI-Centre for Global Health Research (KEMRI-CGHR) with support from the United States Centers for Disease Control and Prevention (US, CDC) ^16^. Pregnancies and their outcomes are regularly (2-3 times-a-year) recorded through active household visits by trained field staff in the three study sites. In Siaya County, additional data were collected from two hospitals, Bondo County Hospital and Siaya County Referral Hospital, leveraging on existing platform for surveillance of influenza disease among pregnant women. This surveillance is also led by KEMRI-CGHR in collaboration with US CDC ^17, 18^.

### Study population

The study population consisted of three different groups of women. The first group comprised of pregnant women who resided in the HDSS area in Kilifi, Siaya (Asembo), and Nairobi, and who were registered as pregnant in the 2017/2018 (Kilifi) and 2018–2020 (Siaya and Nairobi) enumeration rounds. The second group consisted of pregnant women who presented to the maternity wards of Bondo and Siaya hospitals for delivery, between 1 ^st^ February to 30 ^th^ April 2021. The time frame for data collection within the HDSS areas and in the maternity wards allowed us to compare timing for ANC visits before and during the period of the COVID-19 pandemic. At the time of data collection, all participating women had a birth outcome.

The third group consisted of pregnant women who attended ANC clinics at selected health facilities in Kilifi, Siaya, and Nairobi counties during 2019 and whose gestational age was recorded in the hospital ANC registers.

### Data collection

To estimate proportion of pregnant women attending ANC within the proposed gestational age windows for maternal RSV vaccine delivery, data were abstracted from Mother and Child Health (MCH) booklets belonging to women selected from the HDSS areas of Kilifi, Kibera in Nairobi and Asembo in Siaya. Detailed description of the data collection methods has been published ^12^. In brief, in each of the HDSS areas, a computer-generated random sample of 1,000 resident women with a registered pregnancy in 2017–2018 (Kilifi) and 2018–2020 (Kibera in Nairobi and Asembo in Siaya), were selected. They were traced at home by trained field workers for consenting. Upon consenting, data on ANC attendance were abstracted from their MCH booklets. To account for missing women during home visits or those with missing MCH booklets (estimated at 50% ^12^ as experienced in Kilifi HDSS), a replacement sample set of 1,000 women was generated for Nairobi and Siaya, and the lists were uploaded to the computer database.

Additional data on gestational age at ANC attendance were abstracted from MCH booklets of pregnant women presenting for delivery in the two hospitals (Bondo sub-county referral hospital and Siaya County hospitals) in Siaya County upon consenting. This dataset was to provide comparative information on timing for ANC attendance during the COVID-19 pandemic period.

To determine the number of pregnant women attending ANC per day within the proposed gestational age window for maternal RSV vaccine delivery, we conducted an administrative review and abstraction of ANC records of 2019 from selected health facilities in the three counties of Kilifi, Siaya and Nairobi. Purposeful sampling was used ensuring a geographical and facility level representation. Other considerations made were accessibility, population densities of the catchment population and prior involvement of the health facility in other KEMRI projects. A total of 24 health facilities stratified by level of health care dispensaries (level 2), health centres (level 3), sub-county hospitals (level 4) and county referral hospital (level 5) were selected in the three participating counties: ten from Siaya, eleven from Kilifi and three from Nairobi County. The included health facilities by level of care were as follows: five dispensaries, three health centres, two sub-county hospitals, one county referral hospital in Kilifi; three dispensaries, three health centres, three sub-county hospitals, one county referral hospital in Siaya County; and two health centres and one county referral hospital in Nairobi County. Management teams in the 24 selected health facilities were approached by the study coordinators for sensitization and permission sought to access the 2019 ANC registers. Abstraction of ANC timing records from the 24 health facilities started on 1 ^st^ October 2021 and completed on 30 ^th^ April 2022. In all records abstracted, the gestational age at ANC visit was measured by fundal height.

All the abstracted data were entered into a custom-designed electronic database using computer laptops and tablets.

### Statistical analysis

Gestational ages in weeks at ANC visits were presented as mean (Standard Deviation, SD), or median (interquartile range, IQR). The proportions of women attending ANC during two potential gestational age windows for vaccine delivery ( *i.e.*, 28–32 weeks and 24–36 weeks) were calculated as described previously ^12^. We also estimated the mean (SD) and median (IQR) number of pregnant women attending ANC per day during the two vaccination windows stratified by the level of health care and by county. A Bartlett’s test was used for analysis of variance in the average number of daily ANC attendees in all three counties by level of health care. A two-sample t-test was used for analysis of the differences in average number of women attending ANC per day, between two or three counties at different levels of health care. Analysis of the difference in median number of women attending ANC at the three counties stratified by level of health care was conducted using a Mood’s median test. Density curves were used to describe the distribution of gestational age at ANC attendance for data collected from HDSS areas of Kilifi, Asembo in Siaya and Kibera in Nairobi. All analyses were conducted in Stata (RRID:SCR_012763)

 version 15 (Stata Corp, College Station, USA). An alternative statistical software to perform the equivalent analysis, which is open-access, is RStudio version 4.1.1.

### Ethical considerations

Individual written informed consent was obtained from the randomly selected pregnant women residing in HDSS of Kilifi, Asembo in Siaya and Kibera in Nairobi and from all participants presenting to maternity wards of Siaya and Bondo hospitals for delivery. Approval to abstract data on ANC timing from health facilities was granted by the hospital management teams and County departments of Health. This study was approved by the National Commission for Science, Technology and Innovation (NACOSTI License No: NACOSTI/P/21/12896, Dated 15 ^th^ September 2021) and the KEMRI Scientific and Ethical Review Unit (through SERU protocol#: 3716, Dated 16 ^th^ August 2021). The ongoing surveillance in PBIDS obtained ethical approval from SERU (protocol#: 2761, Dated 15 ^th^ September 2015) and CDC Institutional Review Board (IRB) Reliance approval (protocol#: 6775, Dated 15 ^th^ September 2015).

## Results

### Characteristics of participants and participating health facilities

A total of 594 women in Kilifi, 1,029 women in Siaya and 1,079 women in Nairobi were traced, consented and interviewed within the community of the HDSS areas. Out of those traced, 1,025 (99.6%) in Siaya and 1,076 (99.7%) in Nairobi had attended ANC during pregnancy ( Table 1) ^19^. All 594 women in Kilifi reported to have attended ANC during pregnancy. Women with MCH booklets were, 470 (79.1%) from Kilifi as previously described ^12^, 791 (76.9%) from Siaya and 821 (76.1%) from Nairobi, respectively. The median age, in years, of women during pregnancy and at the time of first ANC visit was 28.6 (IQR, 23.4-33.6), 28.4 (IQR, 24.0-32.5) and 29.0 (IQR, 25.0-34.0) years in Kilifi, Siaya and Nairobi, HDSS areas respectively. The median gestational age at ANC initiation was 26 (IQR, 21-28) weeks in Kilifi HDSS, 22 (IQR, 18-26) weeks in Siaya HDSS and 23 (IQR, 19-26) weeks in Nairobi HDSS ( Table 1).

**Table 1.** Characteristics of women selected from the Health Demographic Surveillance System (HDSS) sites of Kilifi, Siaya and Nairobi in Kenya.

Characteristics	Health Demographic Surveillance System Sites		
	Kilifi	Siaya	Nairobi
	N (%)	N (%)	N (%)
Women interviewed (N)	594	1029	1079
Women attended ANC	594 (100.0)	1,025 (99.6)	1,076 (99.7)
Women with MCH * booklets	**470 (79.1)**	**791 (76.9)**	**821 (76.1)**
Median age (IQR) in years	28.6 (23.4-33.6)	28.4 (24.0-32.5)	29.0 (25.0-34.0)
**Education level**			
None	82 (17.5)	1 (0.1)	1 (0.1)
Primary	326 (69.4)	530 (67.0)	411 (50.0)
Secondary	48 (10.2)	231 (29.2)	321 (39.1)
Tertiary-College/University	14 (3.0)	29 (3.4)	88 (10.7)
**Marital status**			
Married	434 (92.3)	720 (91.0)	706 (86.0)
Single	34 (7.2)	49 (6.2)	111 (13.5)
Divorced/Separated/Widowed	2 (0.4)	22 (2.8)	4 (0.5)
**Delivery place**			
Health facility	341 (72.6)	756 (95.6)	802 (97.7)
Home	129 (27.5)	35 (4.4)	19 (2.31)
Proportion attending ANC ** Visit			
ANC1	470 (100)	791 (100)	821 (100)
ANC2	393 (83.6)	725 (91.6)	717 (87.3)
ANC3	286 (60.8)	668 (84.5)	669 (81.5)
ANC4	162 (34.5)	540 (68.3)	540 (65.8)
ANC5	46 (9.8)	322 (40.7)	340 (41.4)
**Median Gestational age at ANC Visit in weeks**			
ANC1	26 (21-28)	22 (18-26)	23 (19-26)
ANC2	29 (26-32)	26 (21-30)	26 (22-30)
ANC3	32 (28-35)	30 (25-34)	30 (26-34)
ANC4	35 (32-36)	32 (28-36)	33 (28-36)
ANC5	36 (34-38)	34 (30-36)	34 (30-37)
**Number and Proportion (%) Attended at Vaccine window**			
28–32 weeks	360 (76.6)	590 (74.6)	547 (66.6)
24–36 weeks	452 (96.2)	709 (89.6)	695 (84.7)

*Mother and Child Health Booklet **Antenatal care (ANC1…ANC5- First antenatal care visit -Fifth antenatal care visit)

A total of 596 women who presented for delivery at the maternity wards of Siaya and Bondo hospitals, consented to participate in the study and were interviewed: 263 in Siaya and 333 in Bondo hospital. All women had attended ANC during pregnancy and had MCH booklets. The median (IQR) age at the time of pregnancy was 24.7 (21.0–29.7) years and the median (IQR) gestational age at ANC initiation was 22 (18–26) weeks ( Table 2).

**Table 2.** Characteristics of participants selected from the maternity wards of Bondo and Siaya county referral hospitals in Kenya, 2021.

Characteristics	Maternity Ward Surveillance Sites		
	Siaya	Bondo	All
	N (%)	N (%)	N (%)
Women interviewed (N)	263	333	596
Women attended ANC *	263 (100)	333 (100)	596 (100)
Women with MCH **booklets	**263 (100)**	**333 (100)**	**596 (100)**
Median age (IQR ***) in years	24.3 (20.5-29.0)	21.3 (24.0-29.8)	24.7 (21.0-29.7)
**Education level**			
**None**	0 (0.0)	1 (0.3)	1 (0.17)
Primary	146 (55.5)	131 (39.3)	277 (46.5)
Secondary	97 (36.9)	145 (43.5)	242 (40.6)
Tertiary-College/University	20 (7.6)	56 (16.8)	76 (12.8)
**Marital status**			
Married	191(72.6)	271 (81.4)	462 (77.5)
Single	68 (25.9)	61 (18.3)	129 (21.6)
Divorced/Separated/Widowed	4 (1.5)	1 (0.8)	5 (0.8)
**Proportion attending each ANC Visit**			
ANC1	263 (100)	333 (100)	596 (100)
ANC2	248 (94.3)	312 (93.7)	560 (94.0)
ANC3	221 (84.0)	250 (75.1)	471 (79.0)
ANC4	159 (60.5)	166 (49.9)	325 (54.5)
ANC5	102 (38.8)	80 (24.0)	182 (30.5)
**Median Gestational age at ANC Visit in weeks**			
ANC1	20 (15-26)	24 (20-27)	22 (18-26)
ANC2	26 (21-30)	27 (23-31)	26 (22-30)
ANC3	28 (25-34)	32 (26-35)	30 (26-34)
ANC4	32 (28-36)	34 (30-36)	32 (30-36)
ANC5	35 (32-36)	36 (32-38)	35 (32-37)
**Proportion Attended at Vaccine window**			
28-32 weeks	190 (72.2)	241 (72.4)	431 (72.3)
24-36 weeks	252 (95.8)	316 (94.9)	568 (95.3)

*Antenatal care (ANC1…ANC5- First antenatal care visit -Fifth antenatal care visit) **Mother and Child Health Booklet ***Interquartile range

In the health facilities’ survey where ANC attendance records were abstracted from hospital registers, a total of 24 health facilities stratified by level of health care had 2019 ANC registers available: 10 from Siaya County, 11 from Kilifi County and three from Nairobi County. Details of the facility levels are provided in Table 3. A total of 62,153 ANC records were abstracted: 33,872 from Kilifi; 19,438 from Siaya and 8,943 from Nairobi. The median age of women at the time of presenting for the first ANC visit in these health facilities was 25 (22–30) years. The median (IQR) gestational age at timing for the first ANC visit was 20 (16–25) weeks ( Table 3).

**Table 3.** Characteristics of pregnant women attending ANC at the 24 selected health facilities in Kilifi, Nairobi and Siaya Counties in Kenya, 2019.

Characteristics	County	Dispensary	Facility Levels		County referral hospital	All facilities
			Health Centre	Sub County hospital		
**Number of facilities**	Kilifi	5	3	2	1	11
	Siaya	3	3	3	1	10
	Nairobi	0	2	0	1	3
	**Total**	**8**	**8**	**5**	**3**	**24**
Number of ANC * records ** abstracted**	Kilifi	4,480	9,945	8,474	10,973	33,872
	Siaya	2,036	4,501	8,068	4,833	19,438
	Nairobi	0	1,493	0	7,350	8,843
	**Total**	**6,516**	**15,939**	**16,542**	**23,156**	**62,153**
Median age (IQR **) in years	Kilifi	25 (21-30)	25 (21-30)	25 (22-30)	26 (22-30)	25 (22-30)
	Siaya	24 (20-29)	25 (21-30)	24 (21-29)	25 (22-29)	25 (21-29)
	Nairobi	-	25 (22-29)	-	27 (23-31)	26 (23-31)
	**All**	**24 (21-30)**	**25 (21-30)**	**25 (21-29)**	**26 (22-30)**	**25 (22-30)**
**ANC initiation Median** ** gestational age in weeks**	Kilifi	22 (18-26)	21 (16-25)	21 (17-25)	21 (16-25)	21 (16-25)
	Siaya	20 (12-24)	20 (12-24)	19 (14-24)	20 (15-26)	20 (13-24)
	Nairobi		20 (14-24)		20 (16-24)	20 (16-24)
	**All**	**21 (16-26)**	**20 (16-24)**	**20 (15-24)**	**20 (16-25)**	**20 (16-25)**
**Proportion attending ANC at** ** vaccination-window 28-32 weeks**	Kilifi	34.1% (1,527)	29.2% (2,907)	26.7% (2,260)	24.7% (2,712)	27.8% (9,406)
	Siaya	28.8% (587)	23.7% (1,068)	24.4% (1,966)	20.2% (975)	23.6% (4,596)
	Nairobi	-	23.0% (343)	-	22.8% (1,679)	22.9% (2,022)
	**All**	**32.4% (2,114)**	**27.1% (4,318)**	**25.6% (4,226)**	**23.2% (5,366)**	**25.8% (16,024)**
**Proportion attending ANC at ** **vaccination-window, 24-36 weeks**	Kilifi	69.0% (3,091)	64.9% (6,453)	60.4% (5,120)	59.4% (6,522)	62.5% (21,186)
	Siaya	61.2% (1,245)	55.2% (2,484)	53.9% (4,348)	50.5% (2,442)	54.1% (10,519)
	Nairobi		60.2% (898)		52.4% (3,854)	53.7% (4,752)
	**All**	**66.5% (4,336)**	**61.7% (9,835)**	**57.2% (9,468)**	**55.4% (12,818)**	**58.7% (36,457)**

*Antenatal care **Interquartile range

### The number of women attending ANC per day by level of healthcare

Only data abstracted from the hospital ANC registers were analysed to provide the number of women attending ANC per day.

The number of women attending ANC per day in the different levels of health care, ranged from median (IQR) of 10 (6–17) in dispensaries (level 2), 16 (17–30) in health centres (level 3), 32 (19–42) in sub-county hospitals (level 4) and 30 (19–44) in county referral hospitals (level 5). Summary of ANC attendance within the three counties at different levels of care is provided in Table 4.

**Table 4.** Number of women attending antenatal care per day in selected health facilities of Kilifi, Siaya and Nairobi counties within the required gestational age window for maternal respiratory syncytial virus (RSV) vaccine delivery in 2019.

County	ANC * Attendance per day	Facility Levels				Sub County hospital		County referral hospital	
		Dispensarcy		Health Centre					
		Mean (SD)	Median (IQR)	Mean (SD)	Median (IQR)	Mean (SD)	Median (IQR)	Mean (SD)	Median (IQR)
**Kilifi**	Number of women per day	17 (9.88)	17 (10-22)	36 (20.56)	34 (23-46)	32 (17.47)	33 (19-44)	42 (16.46)	42 (32-53)
	Number at vaccine window 28-32 weeks	6 (3.94)	6 (3-8)	11 (6.71)	11 (7-14)	9 (5.63)	8 (5-12)	11 (5.35)	10 (6-14)
	Number at vaccine window 24-36 weeks	12 (7.08)	11 (7-16)	24 (13.59)	23 (16-31)	20 (11.50)	19 (12-27)	25 (11.13)	25 (18-32)
**Siaya**	Number of women per day	8 (4.17)	7 (5-10)	17 (8.09)	17 (12-23)	30 (15.09)	32 (19-41)	19 (8.65)	20 (14-26)
	Number at vaccine window 28-32 weeks	3 (1.68)	2 (1-4)	4 (2.73)	4 (2-6)	8 (4.49)	8 (4-11)	4 (2.48)	4 (2-5)
	Number at vaccine window 24-36 weeks	5 (3.01)	4 (3-7)	10 (5.19)	9 (6-13)	17 (9.18)	17 (9-24)	10 (4.94)	10 (6-13)
**Nairobi**	Number of women per day			6 (4.63)	5 (3-9)			37 (19.31)	36 (24-47)
	Number at vaccine window 28-32 weeks			2 (1.63)	2 (1-3)			9 (5.14)	8 (5-12)
	Number at vaccine window 24-36 weeks			4 (3.29)	3 (2-6)			20 (10.28)	19 (13-27)
**All**	Number of women per day	12 (8.93)	10 (6-17)	21 (18.06)	16 (17-30)	31 (16.38)	32 (19-42)	33 (18.21)	30 (19-44)
**Counties**	Number at vaccine window 28-32 weeks	5 (3.53)	4 (2-6)	7 (5.97)	5 (2-9)	8 (5.12)	8 (4-12)	8 (5.30)	6 (4-11)
	Number at vaccine window 24-36 weeks	8 (6.48)	7 (4-12)	13 (12.21)	10 (4-19)	18 (10.49)	18 (10-26)	18 (11.27)	16 (10-26)

*Antenatal care

The frequency of ANC attendance in health centres, sub-county hospitals and county hospitals showed a near normal distribution in all three counties. The size of ANC attendance per day varied by facility levels and county (Bartlett’s test across county referral hospitals; Chi2 p<0.001). Health facilities in Kilifi County registered twice more women attending daily ANC care at the different levels of health care compared to those in Siaya county (Kilifi *vs.* Siaya mean daily ANC attendance: 17 *vs.* 8 in dispensary (t=14.43; p<0.001), 36 *vs.* 17 in health centres (t=13.49; p<0.001) and 42 *vs.* 19 in county referral hospital (t=19.72; p<0.001)). A moods median test also showed the median number of women attending ANC per day in dispensaries in Kilifi and Siaya counties was significantly different (Chi2 p<0.001).

### The number of women attending ANC per day at the proposed vaccination windows and at the different levels of health care

The median (IQR) number of women attending ANC per day at the different levels of health care, who were within the gestational age window of 28-32 weeks in all counties was four (2-6) in dispensaries, five (2-9) in health facilities, eight (4-12) in sub-county hospitals and six (4-11) in county referral hospitals ( Table 4).

Women attending within gestational age window period of 24-36 weeks were seven (4-12) in dispensaries, 10 (4-19) in health centres, 18 (10-26) in sub-county hospitals and 16 (10-26) in county referral hospitals. Health centres in Kilifi registered a median (IQR) of 23 (16-31) pregnant women per day who attended ANC within the vaccine window of 24-36 weeks in 2019 compared to Siaya and Nairobi, which had nine (6-13) and three (2-6) women, respectively Table 4).

### The proportion of women attending ANC at the proposed gestational age window for vaccine delivery

Among women interviewed from HDSS areas and with ANC records abstracted from MCH booklets, 360/470 (76.6%), 590/791 (74.6%) and 547/821 (66.6%) in Kilifi, Siaya and Nairobi, respectively, attended at least one ANC visit within gestational age window of 28-32 weeks. Further analysis showed that, 452/470 (96.2%), 709/791 (89.6%) and 695/821 (84.7%) women in Kilifi, Siaya and Nairobi, respectively, with at least one ANC visit, were within the gestational age window of 24-36 weeks ( Table 1).

For women sampled from the maternity wards and during the COVID-19 pandemic, 190 (72.2%) in Siaya and 241 (72.4%) in Bondo hospital attended at least one ANC visit within gestational age window of 28-32 weeks. The proportion of women who attended at least one ANC visit within gestational age window of 24-36 weeks were, 252 (95.8%) and 316 (94.9%) in Siaya and Bondo hospitals, respectively ( Table 2).

**Table 5.** Proportion of women attending each antenatal care visit by gestational age and at the required window for maternal respiratory syncytial virus (RSV) vaccine delivery, in selected health facilities of Kilifi, Siaya and Nairobi counties in Kenya, 2019.

ANC * Visit	County	Proportion of women attending	Median (IQR) Gest age (weeks)	Women attending at vac- window 28-32 weeks n(%)	Women attending at vac- window 24-36 weeks n(%)
	Kilifi	8,841 (26.1%)	21 (19-25)	1,095 (12.4%)	3,190 (36.1%)
**ANC1**	Siaya	6,475 (33.3%)	20 (13-24)	713 (11.0%)	1,958 (30.2%)
	Nairobi	1,535 (17.4%)	20 (16-24)	138 (9.0%)	374 (24.4%)
	**All counties**	**16,851 (27.1%)**	**20 (16-25)**	**1,946 (11.6%)**	**5,522 (32.8%)**
	Kilifi	7,900 (23.3%)	25 (21-29)	2,162 (27.4%)	4,851 (61.4%)
**ANC2**	Siaya	3,889 (20.0%)	24 (20-28)	892 (22.9%)	2,063 (53.1%)
	Nairobi	1,837 (20.8%)	24 (20-30)	414 (22.5%)	959 (52.2%)
	**All counties**	**13,626 (21.9%)**	**25 (20-29)**	**3,468 (25.5%)**	**7,873 (57.8%)**
	Kilifi	6,716 (19.8%)	28 (24-32)	2,531 (37.7%)	5,087 (75.7%)
**ANC3**	Siaya	3,447 (17.7%)	28 (24-32)	1,139 (33.0%)	2,396 (69.5%)
	Nairobi	1,593 (18.1%)	29 (24-34)	487 (30.6%)	1,030 (64.7%)
	**All counties**	**11,756 (18.9%)**	**28 (24-32)**	**4,157 (35.4%)**	**8,513 (72.4%)**
	Kilifi	5,411 (16.0%)	32 (28-34)	2,028 (37.5%)	4,315 (79.7%)
**ANC4**	Siaya	3,160 (16.3)	31 (27-35)	1,116 (35.3%)	2,362 (74.6%)
	Nairobi	2,019 (22.8%)	33 (28-36)	569 (28.2%)	1,304 (64.6%)
	**All counties**	**10,590 (17.0%)**	**32 (28-35)**	**3,713 (35.1%)**	**7,981 (75.4%)**
	Kilifi	2,791 (8.2%)	33 (29-36)	938 (33.6%)	2,174 (77.9%)
**ANC5**	Siaya	1,413 (7.3%)	33 (30-36)	478 (33.8%)	1,070 (75.7%)
	Nairobi	892 (10.1%)	34 (30-37)	251 (28.1%)	576 (64.6%)
	**All counties**	**5,096 (8.2%)**	**33 (29-36)**	**1,667 (32.7%)**	**3,820 (75.0%)**
	Kilifi	1,357 (4.0%)	34 (30-36)	442 (32.6%)	999 (73.6%)
**ANC6**	Siaya	675 (3.5%)	34 (32-37)	192 (28.4%)	457 (67.7%)
	Nairobi	566 (6.4%)	36 (32-38)	108 (19.1%)	326 (57.6%)
	**All counties**	**2,598 (4.2%)**	**34 (31-37)**	**742 (28.6%)**	**1,782 (68.6%)**
	Kilifi	561 (1.7%)	34 (32-37)	144 (25.7%)	386 (68.1%)
**ANC7**	Siaya	238 (1.2%)	36 (34-38)	44 (18.5%)	135 (56.7%)
	Nairobi	255 (2.9%)	36 (34-38)	37 (14.5%)	118 (46.3%)
	**All counties**	**1,054 (1.7%)**	**36 (32-38)**	**225 (21.4%)**	**639 (60.6%)**
	Kilifi	182 (0.5%)	36 (32-38)	38 (20.9%)	107 (58.8%)
**ANC8**	Siaya	92 (0.5%)	36 (33-38)	15 (16.3%)	47 (51.1%)
	Nairobi	98 (1.1%)	37 (35-38)	12 (12.4%)	45 (45.9%)
	**All counties**	**372 (0.6%)**	**36 (33-38)**	**65 (17.5%)**	**199 (53.5%)**

*Antenatal care (ANC1…ANC8- First antenatal care visit -eighth antenatal care visit)

A summary of records abstracted from ANC registers for the number of women attending each ANC visit within the gestational age window proposed for maternal RSV vaccine delivery is shown in Table 5. The number of women attending first ANC visit within the gestational age window of 28-32 weeks were: 1,095 (12.4%) in Kilifi County, 713 (11.0%) in Siaya County and 138 (9.0%) in Nairobi County. Records of women attending first ANC visit within the vaccination window of 24-36 weeks were: 3,190 (36.1%) in Kilifi County, 1,958 (30.2%) in Siaya County and 374 (24.4%) in Nairobi County ( Table 5). The proportion of ANC attendees with more than five visits was below 5% of the total ANC attendance. Combined data from all counties showed over 70% of ANC attendees presenting for 3 ^rd^ to 5 ^th^ visits were within the vaccination window of 24-36 weeks. At the 8 ^th^ ANC visit, the median gestational age was 36 (33-38) weeks and only 199 (53.5%) of ANC attendees were within the vaccination window of 24-36 weeks with combined data from the three counties ( Table 5).

### Distribution of gestational age at ANC attendance

**Figure 1.**
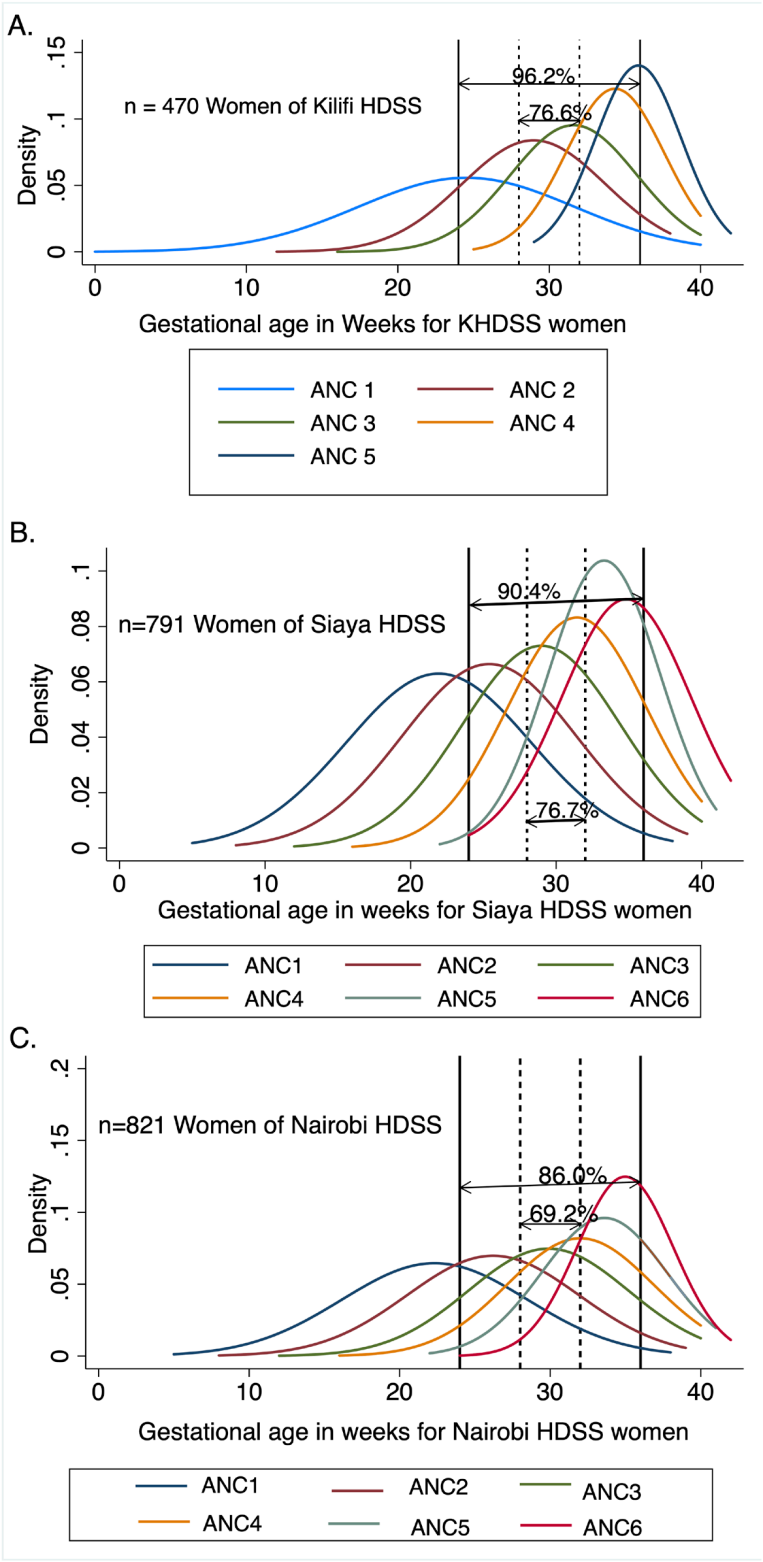
Density distribution curves of gestational age by ANC visit among women sampled from the HDSS areas of Kilifi, Siaya and Nairobi. Each curve represents participant’s ANC visits. Panel A, panel B and panel C, represent density distribution curves of pregnant women for Kilifi, Siaya and Nairobi HDSS areas, respectively. The number of ANC visit (First to Sixth) attended is denoted by ANC1, ANC2, ANC3, ANC4, ANC5 or ANC6. The two gestational age windows (28–32 weeks and 24–36 weeks) for maternal RSV vaccination and the proportion of women attending within that gestational age window for each site are also shown. ANC, antenatal care; HDSS, Health and Demographic Surveillance System; RSV, respiratory syncytial virus.

The distribution of gestational age for women attending ANC sampled from the HDSS areas of Kilifi, Siaya and Nairobi is shown using density curves in Figure 1.

The distribution of gestational age at first ANC visit ranged from a median (IQR) of 20 (16-25) weeks in all counties for data from health facilities ( Table 5) but was delayed in Kilifi HDSS women where it ranged from 21-28 weeks ( Table 1).

## Discussion

In this study, we have presented data on the timing for ANC visits from three counties of Kilifi, Siaya and Nairobi in Kenya. We have shown that, at least 76%, 75% and 67% of pregnant women from Kilifi, Siaya and Nairobi HDSS areas, respectively, attending ANC could be eligible for maternal RSV vaccination if vaccine delivery is at the gestational age window period of 28-32 weeks. Widening the vaccine window to 24-36 weeks of gestational age, will have about 96%, 90% and 85% of women in the HDSS from Kilifi, Siaya and Nairobi, respectively, reached for maternal RSV vaccination at the ANC clinics. Additionally, our results have shown ANC attendance was uninterrupted during the period of COVID-19 pandemic. About 95% of women attending ANC in Siaya and Bondo, during the COVID-19 pandemic would have been eligible for vaccination within the gestational age window of 24-36 weeks. We also found that, within the health facilities, depending on the level of health care, the number of ANC attendees who were within the proposed gestational age window for vaccine delivery ranged from three to six women per day in level 2 health facilities (dispensaries) at the narrow window of 28-32 weeks and to a maximum of 10-25 in level 5 (county referral) hospitals at the wider window of 24-36 weeks across the three counties.

From the findings in this study, we note that, understanding the delivery of maternal tetanus vaccine ^20, 21^ might provide an insight to the vial size for use at ANC clinics for the maternal RSV vaccine. The maternal vaccine in use in Kenya is a bivalent vaccine, which is a combination of Tetanus toxoid-containing vaccines (TTCV) and Diphtheria vaccine (TD vaccine) and is available as a multi-dose vial with 20 doses per vial ^22^. The TD vaccine has a shelf life of 28 days once opened if proper storage (2-8°C) and sterile handling conditions are maintained ^22^. Since TD vaccine does not require restricted timing during its administration, all ANC attendees are eligible for vaccination regardless of gestational age. The basis for implementation of the 20-dose TD vial in this setting also corresponds with the average number of 12 ANC attendees per day in dispensaries, implying usage of the TD vaccine vial for at least two ANC days in the lowest level of health care. For a vaccine requiring restricted gestational age window during its administration, our study has shown an average of five ANC attendees in dispensaries are within the narrow window of vaccination of 28-32 weeks. Assuming all eligible women accept uptake of the maternal RSV vaccine, a multidose vial containing at least five doses may approximate daily ANC attendance and might apply across all levels of care. However, the preferred vaccine vial size might also depend on the shelf life, cost of the vaccine and other factors.

In this study, we found attending more than five ANC visits does not significantly increase the proportion of women who are attending ANC within the proposed gestational age window for vaccination. This is because, by the fifth ANC visit, most ANC attendees were outside the vaccination window of 28-32 weeks. Furthermore, we did not find significant increases in the proportion of women eligible for vaccination if pregnant women attended up to eight ANC visits. We also found at the first ANC visit, about 12% and 33% of the pregnant women were within the proposed vaccination windows of 28-32 weeks and 24-36 weeks, respectively. These findings emphasise the need to start screening pregnant women as early as during the first ANC visit for eligibility of vaccination. On the other hand, increasing the number of ANC visits from the current four as practiced and recommended in the National Guidelines for Quality Obstetrics and Perinatal Care in Kenya ^23^ to eight contacts, which is recommended by the World Health Organization (WHO) ^24^, may be of little benefit in terms of vaccine coverage.

We also found that the proportion of pregnant women presenting in the maternity ward of Siaya and Bondo hospitals who would have been reached for vaccination within the narrow and wider windows during the COVID-19 pandemic were similar with women in HDSS area who attended ANC prior to the pandemic period. These findings are consistent with a study to assess the indirect health effects of the COVID-19 pandemic in Kenya, which did not find any significant change in the utilization of maternal health services ^25^. Although most health care services were disrupted due to SARS-CoV-2 pandemic mitigation measures enforced by the government, not all services were affected, and these included ANC ^26^. This is because, the ministry of health in Kenya issued guidelines ^27^ that prioritized and made essential services such as ANC care, child immunization and delivery services accessible within the health facilities.

In this study we have provided a comprehensive assessment of ANC visit timing among pregnant women in Kenya, but this is not without limitations. We used fundal height, which is not the most accurate measure of gestational age during pregnancy. However, this is what is available and in practice within the public health care system in Kenya. This study only focused on vaccination of pregnant women who attend ANC and has not accounted for those who attend but might decline to take up the vaccine or those not attending ANC at all during pregnancy and how they can be reached for vaccination within the community. Despite these limitations, the results presented can be used to directly infer potential maternal vaccine coverage and provides very important data to guide implementation of maternal RSV vaccines in LMICs.

## Conclusions

In the general population, approximately 70% of pregnant women, attending at least one ANC visit could be eligible for maternal RSV vaccination if delivery is within the window of 28–32 weeks. Prior to and during the COVID-19 pandemic in Siaya county, the timing of the first ANC visit and the proportion of mothers who would have been vaccinated against RSV remained the same. The number of pregnant women eligible for delivery of the vaccine in the health facilities per day varies with level of health care and geographical region. The median number of ANC attendees eligible for maternal RSV vaccination at the wider gestational age window of 24–36 weeks in dispensaries, ranges between 4 to 12 and an average of five women at the narrow window. Therefore, to minimise wastage, a multidose vial size of about five doses per vial might approximate daily ANC attendance in the lower levels of care in such a setting.

## Data Availability

Detailed study data is stored under restricted access and can be made available from the authors upon request through submission of a Data Request Form for consideration by our Data Governance Committee ( dgc@kemri-wellcome.org). Harvard Dataverse: Assessment of gestational age at antenatal care visit among Kenyan women to inform delivery of a maternal respiratory syncytial virus (RSV) vaccine in low- and middle-income countries. https://doi.org/10.7910/DVN/UN6ZCB ^19^. This project contains the following underlying data: ANC vac_window_30062022.tab ANC vial size analysis script_22072022_V1.do ANC_facilitylevel_30062022.tab Kilifi_ANC community dataset_06072022.tab Maternal_ANC timing with booklet_Nairobi.tab Maternal_RSV_Demographic_ANC_Nairobi.tab maternal_rsv_facility_Nairobi_Kilifi_Siaya_22062022.tab maternal_rsv_facility_Nairobi_Kilifi_Siaya_30062022.tab Siaya_ANC community dataset_06072022.tab Siaya_ANC maternity ward dataset_06072022.tab Siaya_Kilifi_combined final dataset_07062021.tab Data are available under the terms of the Creative Commons Attribution 4.0 International license (CC-BY 4.0).
