# Supplementary material for: Assessment of gestational age at antenatal care visits among Kenyan women to inform delivery of a maternal respiratory syncytial virus (RSV) vaccine in low- and middle-income countries
Source: Wellcome Open Res. 2023 Apr 3;8:154. [Version 1] doi: 10.12688/wellcomeopenres.19161.1 (PMC10369009; doi:10.12688/wellcomeopenres.19161.1)
Supplement: Peer review history

# Reviewer report: Reviewer response for version 1

**DOI:** 10.21956/wellcomeopenres.21241.r63796

**Published:** 2023-09-07

**Authors:** Nicholas Geard

**Affiliations:** 1 The University of Melbourne, Melbourne, Victoria, Australia

The reported study extends a prior investigation of attendance at antenatal care in Kilifi County, Kenya to also analyse attendance records in Siaya and Nairobi Counties. The motivation is to assess the likely uptake of a potential maternal RSV vaccine delivered via this setting. The study estimates that approximately 70% of women would attend during the target gestational window. Widening the target window slightly would increase this proportion to ~90%.

 The study is well motivated and reported, and the paper is clearly written.

 I have only relatively minor queries and suggestions. Results from the Kilifi group have already been reported in reference 12 (from the same research team), with some of the tables/figures presenting identical information. While this is acknowledged in the text, it would be helpful to also note in the captions/footnotes of these figures/tables (eg, Fig 1A).

 Given that the Kilifi data was from an earlier time period (2017-2018 vs 2018-2020 for the other Counties) I thought it might have been useful to update the Kilifi group analysis to use 2018-2020 data in order to ensure fairer comparability across the different groups.

 Noting that the three groups represent different geographic locations in Kenya, it might be useful to comment briefly on how representative these are of the broader population. To what extent might the findings from these data be extrapolated? Are there factors that differ across locations that may help explain any observed differences in ANC attendance among the groups?

 Minor point: page 8, left column: "A moods median test" -> "A Mood's median test".

Is the work clearly and accurately presented and does it cite the current literature?

Yes

If applicable, is the statistical analysis and its interpretation appropriate?

Yes

Are all the source data underlying the results available to ensure full reproducibility?

Yes

Is the study design appropriate and is the work technically sound?

Yes

Are the conclusions drawn adequately supported by the results?

Yes

Are sufficient details of methods and analysis provided to allow replication by others?

Yes

Reviewer Expertise:

Mathematical and computational modelling and analysis of epidemiological data.

I confirm that I have read this submission and believe that I have an appropriate level of expertise to confirm that it is of an acceptable scientific standard.

# Reviewer report: Reviewer response for version 1

**DOI:** 10.21956/wellcomeopenres.21241.r56203

**Published:** 2023-07-25

**Authors:** Lisa Noguchi

**Affiliations:** 1 Maternal and Newborn Health, Technical Leadership and Innovations, Jhpiego Corporation, Baltimore, Maryland, USA

This is a well-designed analysis that used review and abstraction of ANC records from registers of 24 health facilities, stratified by level of care, from Kilifi, Siaya, and Nairobi counties. Additional data were drawn from Mother and Child Health booklets of women in Kilifi, Nairobi, and Siaya counties. Authors present descriptive summaries of the number and proportion of women attending ANC within the gestational windows of 28-32 weeks and 24-36 weeks. Authors conclude that, assuming all eligible women accept the maternal RSV vaccine, a multidose vial containing at least five doses may approximate daily ANC attendance and might apply across all levels of care. However, the preferred vaccine vial size might also depend on the shelf life, vaccine cost, and other factors. 

 Comments here are intended solely to encourage consideration of these findings in a broader context of implementation of ANC and rollout/uptake of a new maternal vaccine, and not to detract from or de-emphasize the quality of the analyses. 

 The potential relationship between timing and frequency of ANC contacts and uptake of other ANC interventions with recommended gestational age windows has also been studied for other components of ANC. In previous research, timing and frequency of ANC visits was not significantly associated with odds of getting an ultrasound among pregnant women in Migori County. While obstetric ultrasound is a *very different *intervention in terms of purpose, gestational age window, availability/access, and administration, it is an example of a complex intervention with a wider gestational age window (at least for routine scans) that has yet to achieve its full benefit in low-resource settings, due to gaps in a comprehensive approach to implementation. Ultimately, making an intervention more accessible to ANC clients is necessary but insufficient for optimizing its impact on health outcomes. 

 Based on their findings, the authors propose that increasing the number of ANC visits from the current four as recommended in Kenyan national guidelines to eight contacts recommended by WHO may be of little benefit in terms of vaccine coverage. However, it should be noted that attendance/continuation in ANC and acceptance of ANC interventions are both impacted by many other factors, such as quality of care, particularly client experience of care. Moving to an eight-contact ANC model by itself may not have a significant impact on uptake of a maternal RSV vaccine. However, the 2016 WHO model for ANC goes beyond recommending additional second and third trimester contacts to focus on improving client experience of care, and a comprehensive, high-quality approach to its implementation could have a substantial impact on the available "denominator" of women who attend ANC during the target gestational age window, the nature of client interactions with and trust in ANC providers, and ultimately vaccine uptake. 

 Using these results for planning purposes assumes a somewhat static approach to delivery of ANC services in the coming years. However, localized shifts in how ANC is delivered, e.g., increasing uptake of group-based models to improve continuity in and quality of care; evolution of financing strategies for maternal child health and primary care; and concurrent health system challenges, e.g., projected increases in health worker shortages, may positively or negatively disrupt how and where ANC is delivered in the next decade and beyond - all of which may impact expected gestational age of pregnancies at ANC contacts. 

 In the discussion, the authors appropriately acknowledge factors unable to be addressed by this analysis, including non-attendance at facility-based ANC contacts and vaccine decliners, etc. However, optimal vial size will ideally be just one of multiple strategies to minimize wastage and facilitate high coverage of a maternal RSV vaccine. The authors are appropriately careful to keep their discussions and conclusions reflective of their specific planned analyses. Even so, for those planning for future rollout of maternal RSV vaccines in ANC settings, it may also be useful to consider these results within the larger context of factors that may impact both open and closed vial wastage. 

 Lastly, it should be noted that the proposed "real-world" effectiveness study of the leading maternal RSV candidate vaccine in the next 1-2 years could complement these analyses and also inform optimal vial size with data from a range of countries.

Is the work clearly and accurately presented and does it cite the current literature?

Yes

If applicable, is the statistical analysis and its interpretation appropriate?

Yes

Are all the source data underlying the results available to ensure full reproducibility?

Yes

Is the study design appropriate and is the work technically sound?

Yes

Are the conclusions drawn adequately supported by the results?

Yes

Are sufficient details of methods and analysis provided to allow replication by others?

Yes

Reviewer Expertise:

Clinical trials of HIV pre-exposure prophylaxis for pregnant and breastfeeding populations, observational data analyses of injectable contraception and STI/HIV acquisition, implementation research on improving quality of antenatal and intrapartum care.

I confirm that I have read this submission and believe that I have an appropriate level of expertise to confirm that it is of an acceptable scientific standard.

## References

[1] : Quality of antenatal care and associated factors in a rural county in Kenya: an assessment of service provision and experience dimensions. *BMC Health Serv Res* .2019;19(1) : 10.1186/s12913-019-4476-4 684 10.1186/s12913-019-4476-4 31590662PMC6781384

[2] : Routine antenatal ultrasound in low- and middle-income countries: first look - a cluster randomised trial. *BJOG* .2018;125(12) : 10.1111/1471-0528.15287 1591-1599 10.1111/1471-0528.15287 29782696PMC6390492

# Reviewer report: Reviewer response for version 1

**DOI:** 10.21956/wellcomeopenres.21241.r56130

**Published:** 2023-05-19

**Authors:** Ting Shi

**Affiliations:** 1 Usher Institute, The University of Edinburgh, Edinburgh, Scotland, UK

This is a nice written paper assessing gestational age at antenatal care visits among Kenyan women in order to inform delivery of future maternal respiratory syncytial virus (RSV) vaccine.

 The study population consisted of three different groups of women recruited during different time frames (including before and during the period of COVID-19 pandemic). What was the rational of having different time frames for these three groups of population? Are they comparable? How did COVID-19 affect the recruitment and findings of your study? Was the influence similar across different areas/healthcare settings (e.g., maternity wards vs. health facilities)?

 Since RSV infection is a seasonal illness and it is important to have the vaccine at the right timing. I am wondering if authors have looked at the antenatal care visits by month, whether there is a difference in ANC visits across different months (particularly within RSV season and outside RSV season). A follow-up study on the vaccine hesitancy would be helpful to further understand / plan the delivery of maternal RSV vaccine as even though these pregnant women visited ANC it didn't mean that they would be fine with receiving the vaccines. Also, have you looked at the father's education level and the household size which might also affect the ANC visits among pregnant women?

Is the work clearly and accurately presented and does it cite the current literature?

Yes

If applicable, is the statistical analysis and its interpretation appropriate?

Yes

Are all the source data underlying the results available to ensure full reproducibility?

Yes

Is the study design appropriate and is the work technically sound?

Yes

Are the conclusions drawn adequately supported by the results?

Yes

Are sufficient details of methods and analysis provided to allow replication by others?

Yes

Reviewer Expertise:

respiratory epidemiology, respiratory syncytial virus, global health, data science

I confirm that I have read this submission and believe that I have an appropriate level of expertise to confirm that it is of an acceptable scientific standard.
